# Supplementary material for: Early intervention model for treating mood and anxiety disorders: A realist mixed-methods hypothesis test of emerging adult recovery through the mechanism of agency
Source: PLOS Ment Health. 2024 Jul 19;1(2):e0000008. doi: 10.1371/journal.pmen.0000008 (PMC12798563; doi:10.1371/journal.pmen.0000008)
Supplement: S1 File — A. Protocol. a. Objectives and Aims. b. Methodology. i. Overview. ii. Screening. iii. Intake & Assessment. iv. Data Collection–Quantitative. v. Data Collection–Qualitative. B. Treatment Model. C. References. Patient Satisfaction Questionnaire for FEMAP. (DOCX) [file pmen.0000008.s001.docx]

**SUPPORTING INFORMATION**

1. **PROTOCOL**

**Objectives and Aims**

The long-term goal of our research is to provide effective secondary prevention for youth and young adults with primary mood and/or anxiety disorders with or without other co-occurring conditions. The objective of this research was to evaluate the long-term effectiveness of, and patient satisfaction with, the First Episode Mood and Anxiety Program (FEMAP).

1. To identify the extent to which emerging adults (EAs) within the FEMAP system have a reduction of symptoms related to treatment-as-usual within this novel program. 2) To identify the extent to which youth are satisfied with the treatment they received at FEMAP. 3) To identify the extent to which functional impairment is reduced and quality of health improved by treatment at FEMAP. The logic model of FEMAP is shown in Figure S1.


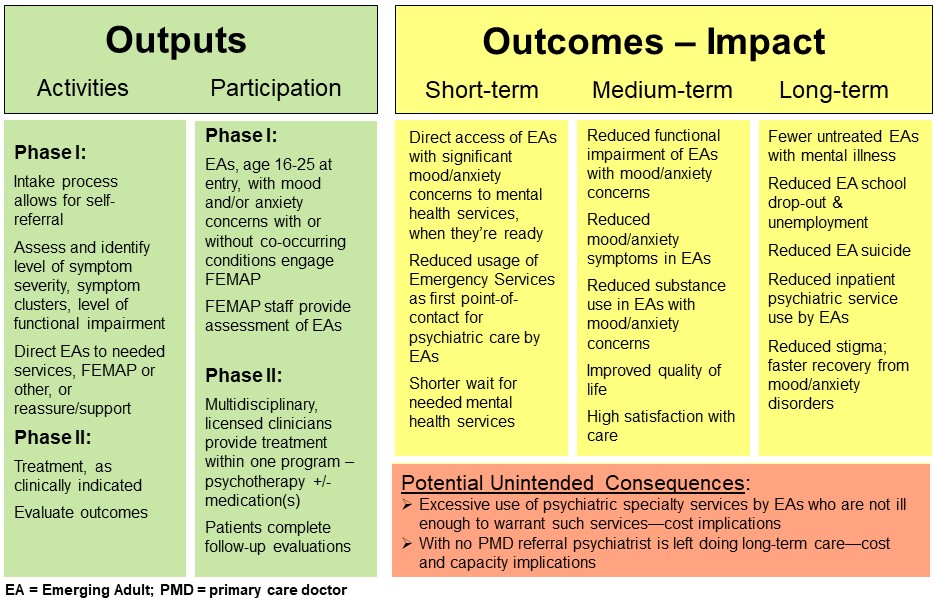
**Figure S1: Logic Model of FEMAP**

**Methodology**

**Overview.** The approach to the above objectives was to conduct a prospective, longitudinal, pre-post evaluation of patients’ symptom severity, overall health care status and functioning before and during/after treatment at FEMAP. Satisfaction with care was also measured.

The optimal design to meet these objectives would have included a control that provided treatment as usual (TAU). However, for ethical reasons we felt unable to utilize a randomized no-intervention control group within FEMAP. It was also impossible, practically, to implement research infrastructure into an existing service to serve as TAU. No such treatment service for EAs existed locally or regionally at the time of this study, with individual family doctors seeing many EAs. Specialized psychiatric child and adolescent services (hospital based and community) stopped service at age 18, and adult services starting at age 18. FEMAP bridges that gap for emerging adults (EAs) aged 16-25 at the time of entry into the program. Thus, TAU involved numerous, unintegrated services and/or individual practitioners, making system evaluation of this approach logistically impossible. We acknowledge that there are threats to internal validity without a comparison design. The interpretation of our results must be somewhat limited by this fact.

Our prior publication of intermediate-term outcomes utilized the wait period between intake and treatment onset for comparison (1) and demonstrated the suitability of this approach for wait list control in the intermediate time-frame. Due to the difference in length of time between the wait control (generally less than 7 months) and the 1-2 years used for the long-term evaluation in this analysis, we did not conduct a comparison of these two epochs.

All data presented in this study were collected prior to the onset of the COVID pandemic declaration in Canada.

**Screening.** The pathway though both care and research at FEMAP is shown in Figure S2 below. All potential patients of FEMAP underwent a brief, 5 question screening process addressing the exclusion criteria. This occurred by telephone directly with the individual wanting care. A parents or other individual was not allowed as a substitute for the phone screening in order to ensure interest of the potential patient for engaging.

All potential patients who passed the screening were given an appointment to meet with a FEMAP Intake clinician/researcher for a more extensive interview, as described below.

Exclusion criteria were: psychiatric medication use history of greater than 18 months, lifetime; current involvement with justice system; major medical condition or illness (e.g., uncontrolled seizures, cancer, multiple sclerosis, uncontrolled diabetes, fibromyalgia, history of traumatic brain injury with loss of consciousness longer than a few minutes); significant learning disability or developmental delay; primary substance use disorder (as determined by the timing of onset of affective symptoms relative to substance use); enrollment in another psychiatric treatment program.

After the first 91 participant/patients were enrolled in the study, it was noted in communications between the Intake clinician and EAs that there was improvement in reported symptoms after the intake. Therefore, a data-collection timepoint was added at the time of screening for all subsequent participant/patients (Figure S2). This allowed for a better evaluation of baseline symptoms and functioning.


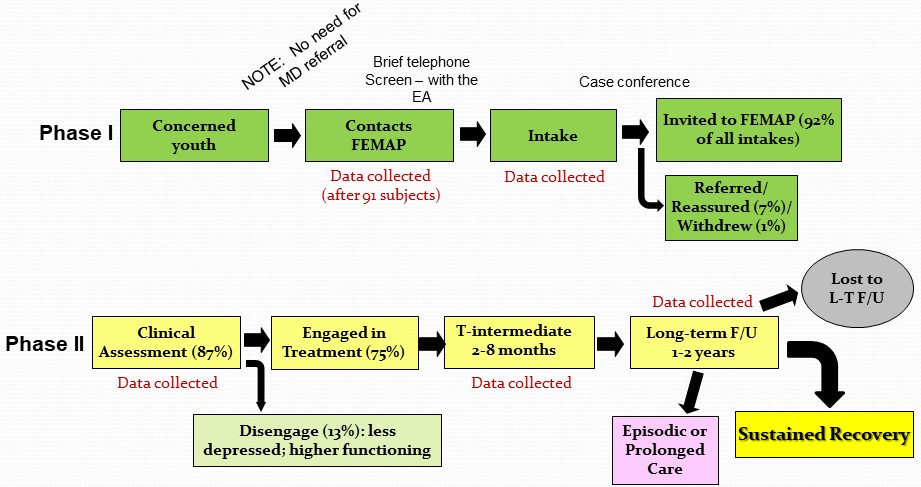


**Figure S2: Pathway through care and research, including possible treatment outcomes.**

Phase I: Initial contact of EA with FEMAP through to decision to accept EA as patient.

Phase II: Treatment course with intermediate- and long-term follow-up.

**Intake & Assessment.** The intake interview was semi-structured, lasted between 60-90 minutes, and was completed in person between the patient and a licensed mental healthcare professional (psychologist, clinical counselor or social worker). This included questions about symptoms, functioning, psychosocial setting, and the potential patients’ goals for treatment and willingness/unwillingness to try medication.

Following the intake, the interviewer/clinician presented the details of the intake to a multidisciplinary case conference of the FEMAP team for discussion about eligibility and treatment planning. Individuals not meeting FEMAP inclusion criteria were either referred to the more appropriate program(s), or reassured if their symptom severity did not seem to require specialized mental healthcare services. All others were accepted into the program.

Following acceptance, patients were placed on a waitlist to see a treatment provider. The default was for the patient to see a psychiatrist, though there were some exceptions (see below). Wait times varied from 2-24 weeks depending on clinician availability. Sometimes this interval lasted longer due to patient choices.

The first appointment involved a comprehensive assessment, after which the clinician and patient developed a treatment plan including psychotherapy goals, as well as discussion about the use of medication. In some cases, either early or later in treatment, patients were referred within the program to our addictions counselor, social worker or family therapist, after discussion of the case with that additional clinician.

Patients who indicated that they were not amenable to using medications, and who also were thought to not require psychiatric services for recovery, per the case conference, were seen by a psychologist, clinical counselor or social worker for psychotherapy, instead of by a psychiatrist. If the patient and clinician together later decided that medication(s) were desired, then they were put on the FEMAP psychiatrist wait list. All non-psychiatrist clinicians on the team had ready access to discussing their cases with team psychiatrists, and vice versa, to facilitate the optimal care of the patients.

**Data Collection - Quantitative.** The schedule of data collection is depicted in the table below.

| **Questionnaire** | **Pre-Intake (Pre-T)** | **Intake assess-ment (TI)** | **Clinical assess-ment** | **Inter-mediate f/u** | **Long-term f/u (TL/T)** |
| --- | --- | --- | --- | --- | --- |
| Demographics and Clinical Summary at entry (bio-psycho-social) |  | X |  |  |  |
| Trauma History Questionnaire (THQ) |  | X |  |  | X |
| Anxiety Sensitivity Index – Revised 36 (ASI) | X | X | X | X | X |
| Montgomery – Asberg Depression Rating Scale – Self-assessment (MADRS) | X | X | X | X | X |
| Patient Satisfaction Questionnaire (PSQ) adjusted for population and service |  | X |  | X | X |
| Quality of Life Enjoyment and Satisfaction Questionnaire-Short Form (Q-LES-Q), scoring modified | X | X | X | X | X |
| Sheehan Disability Scale (SDS) | X | X | X | X | X |
| Brief Cope (BC) |  | X |  | X | X |
| Summary of Services delivered at FEMAP – chart review |  |  |  | X | X |

References for each of the questionnaires appear in the manuscript. The scoring of the Q-LES-Q was modified to remove the question about sex after it was found that the EAs in this sample left that question blank at over twice the rate of any other question, compromising the entire questionnaire score. Additionally, over 50% the subjects left the question about medication use blank, so that question was eliminated from the total score as well.

The Patient Satisfaction Questionnaire used was loosely designed based on Marshall & Hay’s PSQ-18 (2), but heavily modified for a mental health service within a socialized medical care setting (no patient payment required), for the EA age-group. This questionnaire is attached below.

**Data Collection - Qualitative.** The qualitative data reported here were derived from 3 protocols, two of which are published and one is in preparation for publication. The questions from the first of these are associated with a publication by Arcaro et al. (3), and the second of these with a publication by Armstrong et al., (4). The interview question guides for each of these projects can be found at Open Science Framework (OSF), as can the quantitative data set.

**TREATMENT MODEL**

Treatment at FEMAP was conducted by licensed, clinically trained mental health care providers, including psychiatrists, psychologist, social workers and clinical counselors, at various times over the course of this research. Clinicians, including psychiatrists, were trained in, and comfortable practicing, a variety of psychotherapies including but not limited to: cognitive behavioral therapy, motivational interviewing, dialectical behavioral therapy, psychodynamic psychotherapy, interpersonal therapy, graduated exposure and response prevention, trauma treatments (various), and family therapy. Clinician were trained on evidence-based treatment, and yet implemented treatment based on guiding-principles as described below rather than on a predetermined formula or workbook of treatment.

Each of the above psychotherapies was learned in the context of an educational program taken by each clinician in the context of their licensed training program. These instructional materials are structured and, in many ways, formulaic for educational purposes. However, rigid adherence to formulaic approaches with the patients at FEMAP was uncommon because each evidence-based algorithm had to be adapted for patients of varying developmental levels using clinical judgement; algorithms may not apply equally well to any one individual since they do not account for psychosocial variability, nor cultural or cognitive differences. They may prioritize the goals and agenda of the clinician rather than those of the patient, if applied rigidly. Thus, guiding principles were prioritized in treatment, rather than algorithms.

Guiding treatment principles of the treatment model at FEMAP included:

- Developmentally appropriate and individually attuned; clinicians investigate the level of psychosocial development of each EA, and adapt treatment accordingly
- Non-hierarchical within team and with patients (includes social justice principles)
- Build on the patient’s strengths, rather than emphasize their illness
- Prioritize the therapeutic alliance and be partners in care, to include rapport building and mutual agreement on goals and interventions (viz., shared decision-making)
- Remove obstacles to enhance the independence/ problem-solving ability/ agency of patients
- Utilize the patient’s motivation for change, not the clinician’s (alternatively stated: if the clinician is more invested for a particular change than the patient, an adjustment is indicated)

These principles help to inform the alliance with patients because the clinicians are responsive to the patient’s communications and are not working to rigidly impose a specific set of treatment steps onto them.

Clinicians were instructed to use their clinical skills to get the patients “all better” and back to their anticipated developmental trajectory, to the greatest extent possible. Clinicians were expected to use the full extent of their clinical acumen, maximize rapport and therapeutic alliance with the patient and change their treatment approach if the patient was not engaging well or responding to an intervention. The personal goals and aspirations of the patient guided the care, as part of the patient-centered approach.

Model principles were communicated directly and indirectly to clinicians via weekly case conferences which included treatment planning for incoming patients and problem solving regarding existing patients; team meetings that informally addressed the model of care; team retreats (annual or biannual) that reinforced these principles; and research discussions that included clinicians to address clinical research questions within the model of care. Later additions to the model included formal peer supervision occurring monthly, to include all clinicians of all disciplines.

Incoming clinicians and other staff members were interviewed for positions within the program by the Program Lead (EO) and all clinicians were informally monitored for adherence to the principles by all team members. The adherence to the model principles were not systematically monitored or measured, leading to difficulty reporting adherence. However, over the years of the study several clinicians were unable to adjust to the model and either chose to leave or were requested to find employment that better suited their clinical skill sets.

The model recognizes that even non-clinical support staff who interact with patients need to be youth-friendly and patient-focused. The first person to interact with an EA seeking help is often the person answering the phone or meeting them at the door. Youth-friendliness and patient focus is necessary from these support personnel as well.

This framework, using 1) evidence-based psychotherapies applied based on principles rather than formulae or algorithms and 2) a non-hierarchical team structure, maximized the agency of the clinicians, at the process level, for working collaboratively in their role with patients. This first point encourages clinicians to respond in a flexible manner, within the constraints of good clinical practice, in real time, when a patient presents a new treatment goal or therapeutic challenge. The second point encourages team peer interactions across disciplines and level of experience to maximize the collaborative nature and effectiveness of each practitioner and the team as a whole.

This approach maximizes the patient-centeredness of the program in general as well as the treatment intervention with each patient specifically. It makes the preferences and responses of the patient to treatment the priority rather than progress according to an external timeline or mandate by the clinician or service. No maximum number of sessions or frequency of sessions were predetermined. Discharges were generally either bilaterally determined by the client/patient dyad or unilateral on the part of the patient. Very rarely was discharge initiated unilaterally by the clinician.

REFERENCES

1. Osuch E, Vingilis E, Summerhurst C, Demy J, Wammes M, Arcaro J. Process Evaluation of a Treatment Program for Mood and Anxiety Disorders Among Emerging Adults: Preentry Factors, Engagement, and Outcomes. Psychiatr Serv. 2019;70(3):211-8.

2. Marshall GN, Hay RD. The Patient Satisfaction Questionnaire Short-Form (PSQ-18). Santa Monica, CA: RAND; 1994.

3. Arcaro JA, Tremblay PF, Summerhurst C, Wammes M, Dash P, Osuch E. Emerging Adults’ Evaluation of Their Treatment in an Outpatient Mood and Anxiety Disorders Program. Emerging Adulthood. 2019;7(6):432-43.

4. Armstrong S, Wammes M, Arcaro J, Hostland A, Summerhurst C, Osuch E. Expectations vs reality: The expectations and experiences of psychiatric treatment reported by young adults at a mood and anxiety outpatient mental health program. Early Interv Psychiatry. 2019;13(3):633-8.
