# Supplementary material for: Early intervention model for treating mood and anxiety disorders: A realist mixed-methods hypothesis test of emerging adult recovery through the mechanism of agency
Source: PLOS Ment Health. 2024 Jul 19;1(2):e0000008. doi: 10.1371/journal.pmen.0000008 (PMC12798563; doi:10.1371/journal.pmen.0000008)
Supplement: Attachment — Submitted filename: Response to Reviews, 2.docx [file pmen.0000008.s002.docx]

Reviewer #1: Review

Aspects Requiring Additional Evidence

Control of Confounding Variables: Although the study shows significant improvements, the lack of a control group makes it difficult to attribute these improvements exclusively to the treatment model. Future studies with a controlled design would be beneficial to rule out other factors that could influence the results. **Agreed. It is worth noting that we compared our wait list control group with our treatment group in a previous publication of the intermediate-term outcomes using this model/sample. Those findings illustrated that treatment was statistically significantly superior to waitlist control even after the “installation of hope” that might have occurred due to acceptance into a treatment program. We have referenced this article in the present manuscript, which is: Osuch E, Vingilis E, Summerhurst C, Demy J, Wammes M, Arcaro J. Process Evaluation of a Treatment Program for Mood and Anxiety Disorders Among Emerging Adults: Pre-entry Factors, Engagement, and Outcomes. *Psychiatric Services*. 2019;70(3):211-8. The difference in length of time between this wait and the 1-2 year long-term evaluation precluded us from using it as a fair comparison in this analysis.**

**We have added this explanation of the absence of a control group to the Supporting Information section describing the protocol, in the Overview of the methodology section. We have noted the lack of a control group as a limitation of the study in the Discussion. We look forward to the opportunity for more models of early intervention psychiatric care for EAs with mood/anxiety disorders to emerge so that we can compare them with this model.**

Analysis of Subgroups: The study could delve into how different subgroups (for example, based on the severity of the condition, gender, or the presence of concurrent conditions) respond to the treatment. This would help better understand for whom the model is most effective and why. **An excellent idea. We have added a paragraph on *post-hoc* subgroup analyses of our primary outcome that we believed, *a priori*, might have impacted this outcome. We found no effects of sex, trauma exposure category or whether or not the patient was referred by a physician. We did not collect structured diagnostic interviews in this study, so the only metric of diagnostic complexity would be chart review. Given the differences in clinical practice of psychiatric diagnosis, this was deemed unreliable. Additionally, there was such a diversity of concurrent conditions that potential subgroupings would result in small and highly varied subgroup sizes. Thus, we focused on the above-mentioned variables to try to identify subgroups related to outcomes.**

Long-term Follow-up: While the study presents data 1-2 years after the start of treatment, additional research including longer follow-ups could provide valuable information about the durability of the treatment effects. **This is true. Unfortunately, we do not have such data at the present time. We have added a statement to the Discussion section to suggest this for future research.**

Comparison with Other Treatment Models: To further strengthen the study's claims, it would be useful to compare the outcomes of the early intervention model with those of other established treatment approaches. This would help contextualize its effectiveness in relation to available alternatives. **There are many clinical trials of various specific treatments for psychiatric disorders, including for mood and/or anxiety disorders in the EA population. There are very few evaluations of psychiatric service system approaches for the same. We note that comparing the “model” of care at FEMAP to a specific “treatment” for an individual is not evenhanded. It is unfair to compare the model of care at a service to a specific treatment modality used between clinicians and patients, such as cognitive behavioral therapy or the use of fluoxetine plus/minus CBT. (The same way a cancer care program cannot be compared with a specific chemotherapy regime.)**

**As suggested above, it is challenging to compare this model with other models because of the sparsity of such models and since many variables differ, including but not limited to: age of inclusion in the model, location of the model, ethnicity of patients, fee structure to patients, goals of treatment, and others. We have addressed this by adding the following sentences to the Limitations section:**

**“FEMAP is located in a specific region and time, with unique demographics, cultural context and other variable, limiting generalizability of these results. The use of the COMC paradigm in realist evaluation is specifically intended to recognize such influences so that additional iterations of a model may account for such differences. Future studies should investigate the “fit” of the COMC structure described here in other early intervention mood/anxiety programs.”**

Additional Outcome Measures: Incorporating additional outcome measures, such as cognitive functioning or biomarkers of stress, could provide a more holistic understanding of how the treatment affects patients. **Yes, this is true. It would be wonderful if we had such data, but we do not. We appreciate this suggestion and hope to incorporate it into future studies. We have added a statement of such in the Discussion.**


5. PLOS Mental Health encourages authors to publish detailed protocols and algorithms as online supporting information. Does any particular method used in the manuscript warrant such treatment?

Given the innovative nature of the study and the focus on personalized and patient-centered treatment, several aspects of the method used justify the publication of detailed protocols and algorithms as online supporting information, in line with the recommendations of PLOS Mental Health. **We have added a Supporting Information section that provides these details. Thank you for the suggestion.**

Methods Justifying Detailed Publication:

Mixed Realist Evaluation: The study employs a mixed realist evaluation approach to understand how, why, and in what circumstances the treatment program produces its effects. Given the complexity of this approach, which attempts to unravel the interactions between context, mechanism, and outcomes, a detailed protocol would aid other researchers in replicating or adapting the study in different contexts or populations. **We have added a Supporting Information section to describe our protocols/methods in more detail. It should be noted that the use of the realist evaluation model occurred after the data were already collected, which is why our Title and Methods describe the study as applying a realist evaluation hypothesis test rather than posing the study as a realist evaluation complying with the rigorous criteria set forth by the RAMESES II project. In future, this more rigorous standard can be applied.**

Personalized Treatment Focused on Agency: The implementation of a treatment model that focuses on improving the individual agency of patients through a personalized and holistic approach is novel. Publishing detailed algorithms of how treatment is personalized for each patient, including shared decision-making and adaptation of interventions based on the specific needs and circumstances of the patient, would be invaluable to the research community and clinical practice. **We stress that the model uses principles to guide treatment, rather than algorithms which, by definition, are difficult to make responsive to the individual patient in real time. We have pointed out that algorithms or treatment formulae are created in a specific group of treatment recipients who may or may not differ in significant ways from the patient in front of a clinician – thereby compromising the appropriateness of the algorithm (lines 217 to 225 of revised manuscript).**

**In addition, we have added a much more detailed description of the treatment principles used within the model in the Supporting Information section. We greatly appreciate this opportunity to better clarify the method/procedures used in the treatment of patients so that the model can be replicated.**

Mixed Methods of Data Collection and Analysis: The study combines quantitative and qualitative data to assess the treatment program. Detailing the protocols used for collecting, analyzing, and integrating these mixed data would provide crucial guidance for future studies seeking to adopt a similar approach, ensuring consistency and replicability. **Agreed. Again, the data were reviewed for this study retrospectively from other research that was conducted within the program. We have made clear that the interview guidelines for the 3 qualitative projects are available from Open Science Framework.**

Outcome Measures and Evaluation Tools: Publishing details about the specific tools and scales used to measure treatment outcomes, as well as any adaptations made for this particular study, would help other researchers and clinicians better understand the effects of the treatment and how to effectively measure them. **We have added a table to the Supporting Information section that detail the tools used and the timeline, along with modifications to the tools, one of which is now part of the Supporting Information.**

Management of Protocol Deviations: If there were deviations from the original protocol, such as adjustments in the treatment approach due to the COVID-19 pandemic or changes in recruitment strategies, providing a detailed description of these deviations and their justifications would help to contextualize the results and offer lessons learned for future research. **The data reported here were all collected prior to the declaration of the COVID pandemic in the location of the data collection. This is in the paragraph of the Methods that reports on the Ethics procedure (line308).**


Strengths of the Study:

Innovation and Relevance: The study addresses a critical need in the field of mental health, using an innovative approach to the treatment of mood and anxiety disorders in young adults. The importance of developing effective early interventions is well-recognized, and the focus on individual agency as a mechanism of recovery adds a new perspective to the existing literature.

Use of Mixed Methods: The adoption of a mixed-methods design is a significant methodological strength, allowing for a richer and more nuanced understanding of the effects of the early intervention program. The combination of quantitative and qualitative data offers a holistic view of the treatment's efficacy.

Potential Contributions to Clinical Practice: Preliminary findings suggest significant improvements in functioning, symptom reduction, quality of life, and treatment satisfaction, indicating that the intervention model could have valuable practical implications for mental health care.

**Thank you. We greatly appreciate the careful and thoughtful review of our manuscript and your description of its strengths.**

Areas for Improvement:

The manuscript could benefit from greater clarity in the description of the methods employed, including the study design, participant selection, and data analysis. A more detailed justification of methodological choices would also strengthen the manuscript. **We have now provided this in the Supporting Information section.**

A deeper discussion of the study's limitations, including the lack of a control group and how these limitations affect the interpretation of the results, would be valuable. **Added to the Limitations section, as mentioned above, and also in the Supporting Information section.**

Expanding the discussion on how the findings compare and contribute to the existing literature would help position the study within the broader field of mental health research. **We note that realist evaluations of EA mental health treatment programs are scarce (we could find none that addressed psychiatric treatment for mood and/or anxiety disorders). We have acknowledged this and added a paragraph to the Discussion to reference some published protocols from which future results may be expected.**

Providing explicit recommendations for future research could guide further development in this area. **We have added to the Limitations section to include more on future directions for research.**

The authors must ensure that any data shared complies with privacy regulations and informed consent.

**We have consulted with the relevant Ethics Review Board and have now clarified that we are able to post the data at Open Science Framework, which we have now done.**

9. "Are the methodological details sufficient to allow for the replication of the experiments?

The study's methodology requires more clarity, particularly in the customization of treatment and the implementation of shared decision-making. Although mixed methods are used, the lack of detail in the collection and analysis of qualitative and quantitative data hinders a complete understanding and replication of the study. Furthermore, justification is required for the choice of a 20% threshold for the imputation of missing data and examination of other data management techniques. Lastly, it is essential to provide more detailed descriptions of the intervention and its procedures to ensure adequate replicability. In summary, the current methodology is not detailed enough to permit an accurate reproduction of the experiments."

**We have added details of the protocol and data collection in the Supporting Information section.**

**Thank you for catching this detail regarding data missing completely at random. The 20% was a typographical error. The threshold was 10% and we have now corrected that.**


11."Is the manuscript well organized and clearly written to be accessible to non-specialists?

The manuscript is well organized and written with the necessary clarity to be accessible to non-specialists. It strikes a balance between technical rigor and readability, avoiding excessive jargon and explaining technical terms as they are introduced. The structure of the manuscript is logical and coherent, with a clear introduction followed by well-defined sections for methods, results, and discussion, as well as a concise summary and conclusions emphasizing practical implications. Data presentation is facilitated through the effective use of clear and well-labeled tables, figures, and graphs. The manuscript makes relevant connections with broader issues and practical applications, helping to contextualize the research. For orientation towards the non-specialist reader, the abstract and keywords are clear and relevant, the introduction sets out the research problem and its importance in an accessible way, the discussion connects findings with their practical implications in an understandable manner, and the conclusion highlights key points and their relevance to a broad audience." **Thank you for your positive description of our manuscript.**

Reviewer #2: 

3. Data and Analysis Support: The data and analysis largely support the study's claims. However, it could benefit from additional evidence in certain aspects, such as control of variables and detailed analysis by subgroups. **We have added a paragraph on *post-hoc* subgroup analyses of our primary outcome that we believed, *a priori*, might have impacted this outcome. We found no effects of sex, trauma exposure category or whether or not the patient was referred by a physician. We did not collect structured diagnostic interviews, so the only metric of diagnostic complexity would be chart review. Given the differences in clinical practice of psychiatric diagnosis, this was deemed problematic. Additionally, there was such a diversity of concurrent conditions that potential subgroupings would result in small and highly varied subgroup sizes. Thus, we focused on the above-mentioned variables to try to identify subgroups related to outcomes.**


5. Publication of Protocols and Algorithms: Detailed publication of protocols and algorithms, especially on the mixed realist evaluation and personalized treatment focused on agency, would be beneficial. **Thank you for the opportunity to include these. We have now included a Supporting Information section for this manuscript that provides more detail on both the protocol and the model of treatment.**

6. Potential for Resubmission of a Revised Version: Given its significant potential, the authors are encouraged to improve and consider resubmitting a revised version, focusing on greater methodological specificity and discussion of limitations. **We have added to the details of the method in the manuscript itself and we have added a Supporting Information section. The manuscript Limitations section has been expanded per this recommendation.**

7. Original Data Depository: Although the data are not publicly available due to privacy concerns, there is the possibility of accessing a partially redacted version under ethical criteria. **We have checked with our Research Ethics Board and discovered that it is acceptable to make the de-identified data associated with this study available. These have now been deposited at Open Science Framework and are available.**


9. Sufficiency of Methodological Details: Greater clarity is required in the methodology, especially in treatment customization and shared decision-making. **We stress that the model uses principles to guide the evidence-based treatment, rather than algorithms. It is, therefore, difficult to precisely define how treatment is customized. However, we have further defined the treatment model in the Supporting Information section and we believe this information provides sufficient guidance to replicate the model.**

**We have added to the Methods section where we describe the intervention as well, and refer to the Supporting Information for more detail.**
